# Reply: Predictive factors for severe toxicity of sunitinib in unselected patients with advanced renal cell cancer

**DOI:** 10.1038/sj.bjc.6605304

**Published:** 2009-09-15

**Authors:** A A M van der Veldt, E Boven, A J M van den Eertwegh, J B A G Haanen

**Affiliations:** 1 Department of Medical Oncology, VU University Medical Center, Amsterdam, The Netherlands; 2 Department of Medical Oncology, The Netherlands Cancer Institute, Antoni van Leeuwenhoek Hospital, Amsterdam, The Netherlands

**Sir,**

We thank Dr Rezaei Kalantari for his interest in our study. Dr Rezaei Kalantari describes two patients with side effects requiring dose reduction of sunitinib, but who appeared to have developed an improved tolerance to sunitinib after re-escalation of the dose. As the dosing schedule of sunitinib is fixed as 50 mg day^−1^ for 4 weeks on and 2 weeks off regardless of the differences in pharmacokinetic behaviour between individuals, it may be expected that some patients tolerate higher doses, whereas others need dose reduction (Van der Veldt *et al*, 2008). We agree that dose escalation needs to be considered in patients without side effects when aiming for a tumour response. The probability of a greater response and survival increases with higher drug exposure in metastatic renal cell cancer, although the survival of patients treated with lower sunitinib doses on a continuous daily base overlaps with that of patients treated with standard dosing (Escudier *et al*, 2009). The two cases mentioned by Dr Rezaei Kalantari are indeed interesting, because dose reduction was initially required because of a series of side effects. Unfortunately, it is not mentioned whether baseline disease symptoms in these patients aggravated the adverse events. Patients recovered in cycles 3–4, suggesting that adverse events not only disappeared by reducing the dose but also because of a clinical response to sunitinib. It is also not clear whether the patients were taking co-medication that could have interfered with pharmacokinetics of sunitinib. Sunitinib is metabolised primarily by the cytochrome P450 (CYP) 3A4 isozyme to the active metabolite SU012662, which is further metabolised by CYP3A4 (Adams and Leggas, 2007). The parent compound and active metabolite reach similar plasma concentrations and have similar biochemical activity and potency. Besides the well-known inhibitors and inducers of CYP3A4, many other drugs may affect the drug metabolism by CYP3A4. For example, statins have been shown to increase CYP3A expression *in vitro* (Willrich *et al*, 2009). Thus far, no evidence of autoinduction of sunitinib or SU012662 metabolism has been observed with prolonged dosing. As mentioned earlier, measurements of plasma levels of sunitinib and its active metabolite may be of value to determine the dose of sunitinib in particular individuals, such as in obese patients (Desar *et al*, 2009). For the time being, because sunitinib plasma levels cannot be determined routinely, it seems plausible to consider dose re-escalation in fit patients upon signs of disease progression.
